# Regulation of 4E-BP1 activity in the mammalian oocyte

**DOI:** 10.1080/15384101.2017.1295178

**Published:** 2017-03-08

**Authors:** Denisa Jansova, Marketa Koncicka, Anna Tetkova, Renata Cerna, Radek Malik, Edgar del Llano, Michal Kubelka, Andrej Susor

**Affiliations:** a Institute of Animal Physiology and Genetics, ASC, Libechov, Czech Republic; b Institute of Molecular Genetics, ASCR, Prague, Czech Republic

**Keywords:** 4E-BP1, CDK1, cumulus cells, kinase, mTOR, mRNA, meiosis, oocyte, spindle, translation

## Abstract

Fully grown mammalian oocytes utilize transcripts synthetized and stored during earlier development. RNA localization followed by a local translation is a mechanism responsible for the regulation of spatial and temporal gene expression. Here we show that the mouse oocyte contains 3 forms of cap-dependent translational repressor expressed on the mRNA level: 4E-BP1, 4E-BP2 and 4E-BP3. However, only 4E-BP1 is present as a protein in oocytes, it becomes inactivated by phosphorylation after nuclear envelope breakdown and as such it promotes cap-dependent translation after NEBD. Phosphorylation of 4E-BP1 can be seen in the oocytes after resumption of meiosis but it is not detected in the surrounding cumulus cells, indicating that 4E-BP1 promotes translation at a specific cell cycle stage. Our immunofluorescence analyses of 4E-BP1 in oocytes during meiosis I showed an even localization of global 4E-BP1, as well as of its 4E-BP1 (Thr37/46) phosphorylated form. On the other hand, 4E-BP1 phosphorylated on Ser65 is localized at the spindle poles, and 4E-BP1 phosphorylated on Thr70 localizes on the spindle. We further show that the main positive regulators of 4E-BP1 phosphorylation after NEBD are mTOR and CDK1 kinases, but not PLK1 kinase. CDK1 exerts its activity toward 4E-BP1 phosphorylation via phosphorylation and activation of mTOR. Moreover, both CDK1 and phosphorylated mTOR co-localize with 4E-BP1 phosphorylated on Thr70 on the spindle at the onset of meiotic resumption. Expression of the dominant negative 4E-BP1 mutant adversely affects translation and results in spindle abnormality. Taken together, our results show that the phosphorylation of 4E-BP1 promotes translation at the onset of meiosis to support the spindle assembly and suggest an important role of CDK1 and mTOR kinases in this process. We also show that the mTOR regulatory pathway is present in human oocytes and is likely to function in a similar way as in mouse oocytes.

## Introduction

Translational control of specific mRNAs is a widespread mechanism of gene regulation and contributes to diverse biologic processes in many cell types. During the meiotic division of mammalian oocytes (so called oocyte maturation) protein synthesis plays an important role in controlling the progress of meiosis, since the regulation of gene expression on the level of transcription is ceased. At the onset of the first meiotic division, nuclear envelope breakdown (NEBD; G2/M transition) occurs, chromosomes condense and a bipolar spindle forms from the microtubule organizing centers.^1^ During meiosis I, the spindle migrates from the center of the oocyte to the cortex, and the oocyte undergoes an asymmetric division resulting in a large egg competent for fertilization and a relatively small polar body. Proper positioning of the spindle during asymmetric cell division ensures correct partitioning of cellular determinants.^2^ How these events are orchestrated in detail remains unclear.

The importance of protein synthesis for meiotic and mitotic progression has been shown previously. Those published results revealed that protein synthesis is not required for NEBD in mouse oocytes, although the formation of the spindle and progression to metaphase II requires active protein synthesis.^3^ In contrast, positive regulators of the cap-dependent translational pathway become activated post NEBD and inactivated after fertilization.^4-9^

Regulation of translation occurs mainly at the initiation step, which was shown to be rate limiting for overall protein synthesis.^10^ Protein factors that bind to the cap structure at the 5′UTR (untranslated region) and to the 3′UTR-poly(A) sequence of mRNAs have been identified as being essential for this process. Most of the interactions of these proteins are regulated by phosphorylation.^11,12^ The best described protein kinase regulating translation initiation is the mTOR/FRAP kinase, the targets of which are the Eukaryotic initiation factor 4E-binding protein 1 (4E-BP1)^13^ and the S6 kinase.^14^ Hypo-phosphorylated 4E-BP1 binds eIF4E and in such a way inhibits the formation of a translation initiation complex (eIF4F) at the cap structure. EIF4F contains eIF4E (the cap-binding protein), eIF4G1 (the scaffold protein) and eIF4A (an RNA helicase). This complex is probably critical for the translation of mRNAs with extensive secondary structure in their 5′UTR. Upon resumption of meiosis, 4E-BP1 becomes phosphorylated at several sites resulting in its release from eIF4E, allowing eIF4F formation. Phosphorylation at Ser65 and Thr70 modulates the binding of 4E-BP1 to eIF4E directly. Phosphorylation of these sites depends upon 4E-BP1s C-terminal TOR signaling motif that binds Raptor, a component of the mTORC1. Phosphorylation at Thr37/46, which is known to be mediated by mTOR, is required for the modification of Thr70 and Ser65, reflecting the hierarchical phosphorylation of 4E-BP1,^15^ and depends upon 4E-BP1s N-terminal RAIP motif.^16^ Phosphorylation of Thr37/46 is profoundly inhibited by starving cells of amino acids, which inactivates mTOR signaling.^17^ mTORC1 signaling is activated via phosphatidylinositide 3-kinase and protein kinase B (PKB, also termed AKT) and by the Ras/Raf/ERK pathway.^18^ AKT plays a substantial role during the progression of meiosis from GV-stage (germinal vesicle – nucleus in the oocytes) to the MI/MII-stage.^19,20^ Involvement of the mTOR/4F axis in translational regulation during mitosis might be used as a model case for the meiotic cell. Increased phosphorylation of 4E-BP1 has been detected during the meiotic progression of mammalian oocytes,^4,21,22^ and different phosphorylated forms of 4E-BP1 have been shown to co-localize with the meiotic spindle in mouse oocytes.^9,22^ Blocking of 4E-BP1 phosphorylation during maturation has also resulted in the irreversible arrest of metaphase I in bovine oocytes,^23^ abnormal formation of MII spindles in mouse oocytes^9^ or affected asymmetric division.^24^

The aim of this work was to study the metabolic pathways which are involved in 4E-BP1 phosphorylation during in vitro meiotic maturation of mouse oocytes. We discovered that 4E-BP1 becomes phosphorylated in post-NEBD stage oocytes and this phosphorylation remains constant until the MII stage of oocyte maturation and promotes specific translation, which affects spindle assembly. Furthermore, we have uncovered the involvement of different kinases which are potentially involved in the phosphorylation of 4E-BP1.

## Results

### Only 4E-BP1 is present in the mouse oocyte

**Figure 1.**
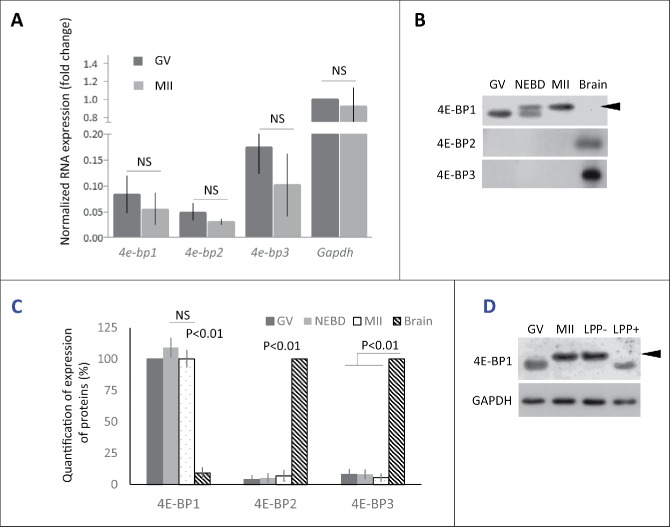
Expression of 4E-BP forms in mouse oocytes. (A) Quantitative RT-PCR analysis shows all 3 forms of *4e-bp* mRNA, which are stable during oocyte maturation (NS = non-significant, n ≥ 3). Results were normalized to the relative internal standard *Gapdh* mRNA in GV. (B) Immunoblotting shows presence of only 4E-BP1 form on the protein level. Both 4E-BP2 and 4E-BP3 are absent in the oocytes, although they are present in the brain. Expression of the 4E-BP1 in the brain sample is significantly lower in comparison with oocytes (See Fig. S1A). 4E-BP1 displays visible phosphorylation shift (arrowhead) post NEBD (a typical experiment from at least 3 replicates is shown). (C) Quantification of protein expression of the 4E-BP1–3 in the oocytes during maturation and brain samples. Data are presented as mean ± SD, Student's t-test. (D) Treatment of the lysate from MII oocytes with Lambda Protein Phosphatase (LPP+) suppressed mobility shift of the 4E-BP1 on the WB. Arrowhead points to phospho 4E-BP1 form. See Figure S1A and B.

In mammals 3 genes code 4E-BP1, 2 and 3.^25^ Our first objective was to determine which form is dominant during mouse oocyte meiotic maturation from the GV to MII stage on the mRNA level. Quantitative RT-PCR analysis showed the presence of all 3 forms of *4e-bps* but with a slightly higher abundance of *4e-bp3*. The global amount of the mRNAs for the 3 different *4e-bps* remained constant throughout meiosis from GV to MII oocytes (Fig. 1A).

Next, we analyzed the presence of all 3 isoforms on the protein level. Our WB analyses showed an absence of 4E-BP2 and 4E-BP3 proteins in the oocytes, which is in the contrary to the results obtained from WB analyses of brain lysate (Fig. 1B and C). However, 4E-BP1 was highly abundant in mouse oocytes with an increased mobility shift post-NEBD (Fig. 1B). Our data showed higher presence of the 4E-BP1 protein in the oocytes than in the brain sample (Fig. 1B and C; Supplementary Fig. 1A). WB also showed that whole population of 4E-BP1 in MII stage oocytes is present as the upper (presumably phosphorylated) band. Treatment of MII oocyte lysate with lambda protein phosphatase (LPP) resulted in the disappearance of the upper band and mobility shift toward lower band, similar pattern to that seen in the GV stage oocytes (Fig. 1D). The experiment in the Fig. 1D shows that mobility shift represents phosphorylation of the 4E-BP1. Moreover, the appearance of mobility shift was confirmed by microinjection of oocytes with RNA coding for 4E-BP1 protein tagged with hemagglutinin (HA). The oocytes were kept in the GV stage or matured for 3 h to NEBD and to MII for 12 h and analyzed by WB, using HA antibody. Our data showed no phosphorylation shift in the GV oocytes, appearance of 2 bands in the NEBD oocytes and whole expressed exogenous HA-4E-BP1 was phosphorylated in the MII stage (Supplementary Fig. 1B).

**Figure 2.**
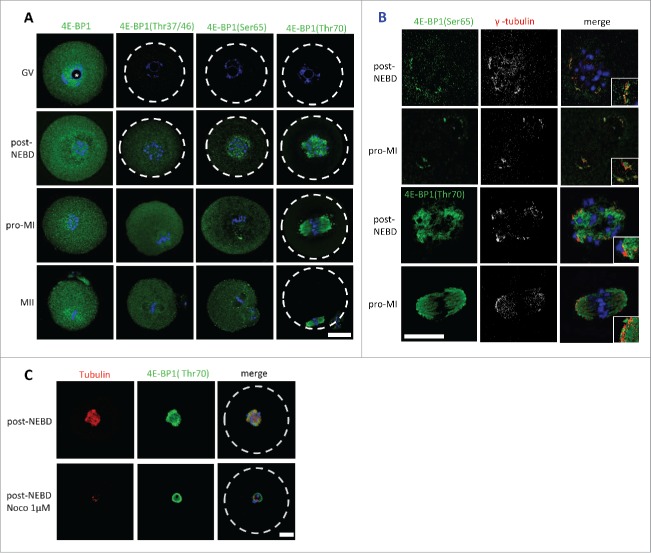
Localization of 4E-BP1 and its phosphorylated forms in the oocytes. (A) Confocal images of different meiotic stages GV (germinal vesicle), post-NEBD (3 h post IBMX wash, PIW), pro-MI (7 h PIW) and MII (12 h) stained with phospho-specific antibody (green) and DAPI (blue), white line indicates oocyte edge. Scale bar = 25 µm. Nucleolus is depicted by asterisk, from at least 3 replicates and n ≥ 30. (B) Marker of the microtubule-organizing centers, gamma tubulin (pseudo-colored and red) co-localizes with 4E-BP1 (Ser65) and (Thr70). Scale bar = 25 µm, n = 10. Enlarged detail in the right bottom corner. (C) Confocal images of control oocytes and oocytes treated with 1 µM Noco for 1 h in the post-NEBD stage (n ≥ 28), tubulin (red), 4E-BP1 (green) and DNA (blue). Scale bar = 20 µm.

It is well established that phosphorylation of 4E-BP1 plays an important role in the regulation of cap-dependent translation.^25-30^ We thus investigated the localization of 4E-BP1 and its phosphorylated forms (Thr37/46/70 and Ser65) during meiosis I. We analyzed different meiotic stages of maturing oocytes; a germinal vesicle (nucleus is present, prophase I) stage was collected directly post isolation; oocytes underwent NEBD following release from the 3-Isobutyl-1-methylxanthine (IBMX) block, oocytes undergone naturally NEBD within 1 h, a group post-NEBD was collected 3 h post IBMX wash (PIW); a metaphase I (MI) stage was collected 7 h PIW and metaphase II (MII) oocytes were collected 12 h PIW. Cell cycle progression was monitored by timing and by immunocytochemistry (ICC) using DNA staining with DAPI. Pan 4E-BP1 antibody was used to analyze the localization of global 4E-BP1 during GV to MII (Fig. 2A). In GV oocytes global 4E-BP1 was evenly distributed throughout the cytoplasm but with a higher signal visible in the nucleoplasm (Fig. 2A and Supplementary Fig. 3), without staining in the nucleolus (marked by asterisk). In the post-NEBD stages the global 4E-BP1 was also spread evenly with just a slight increase at the spindle. ICC experiments using phospho-specific antibody against the Thr37/46 form showed no signal in the GV and a similar localization was seen as total 4E-BP1 protein in the post-NEBD. Antibody recognizing 4E-BP1 phosphorylated at Ser65 showed an increased fluorescence signal in the vicinity of chromosomes, at the spindle assembly area and later at the spindle poles. The pattern of 4E-BP1 phosphorylation at Thr70 showed significant localization at the newly forming spindle post-NEBD or bipolar spindle at MI and MII, and was also present in the extruded polar body. The phospho-specific antibodies did not show a positive signal in the GV stage, which is in a good agreement with our WB data (Fig. 1B and Supplementary Fig. 1A, B). Moreover, double staining of 4E-BP1 phosphorylated at Ser65 or Thr70 with marker of microtubule organizing centers γ-tubulin showed significant enrichment of the 4E-BP1(Ser65) signal in the region with stained γ-tubulin; however, 4E-BP1(Thr70) was distributed along the whole spindle (Fig. 2B).

As 4E-BP1 phosphorylated at the Thr70 was found to be exclusively localized at the forming spindle, we therefore speculated whether this localization was tubulin-dependent. We disrupted the spindle by treatment with 1 µM Nocodazole (Noco) for 1h post-NEBD. Although the dissolved spindle changed the 4E-BP1 (Thr70) pattern, the fluorescence signal still persisted at the chromosomal area (Fig. 2C).

### Activity of mTOR is increased in the human oocyte post-NEBD

As the mouse oocyte is a model organism for the study of human oocytes, we speculated whether mTOR(Ser2448) in human oocytes would be activated similarly as in the mouse oocyte, with a comparable localization pattern. ICC staining of human oocytes in GV, NEBD and MII stages showed that there was no signal for phospho-specific antibody against mTOR(Ser2448) in the GV stage (Supplementary Fig. 3) but increased fluorescence was visible in the NEBD and MII stage. The MII oocyte produced normally formed spindle stained with anti-tubulin antibody with a strong signal for mid-body structure positive for mTOR(Ser2448) (Supplementary Fig. 3).

### 4E-BP1 phosphorylation requires mTOR and CDK1 activity

The timing of increased phosphorylation of 4E-BP1 positively correlates with increased cap-dependent translation after NEBD in the mouse, porcine and bovine oocyte.^5,9,21^ Also, the timing of the increased phosphorylation of mTOR after NEBD.^9,31^ suggests a potential role for mTOR in 4E-BP1 phosphorylation during mammalian meiosis.

**Figure 3.**
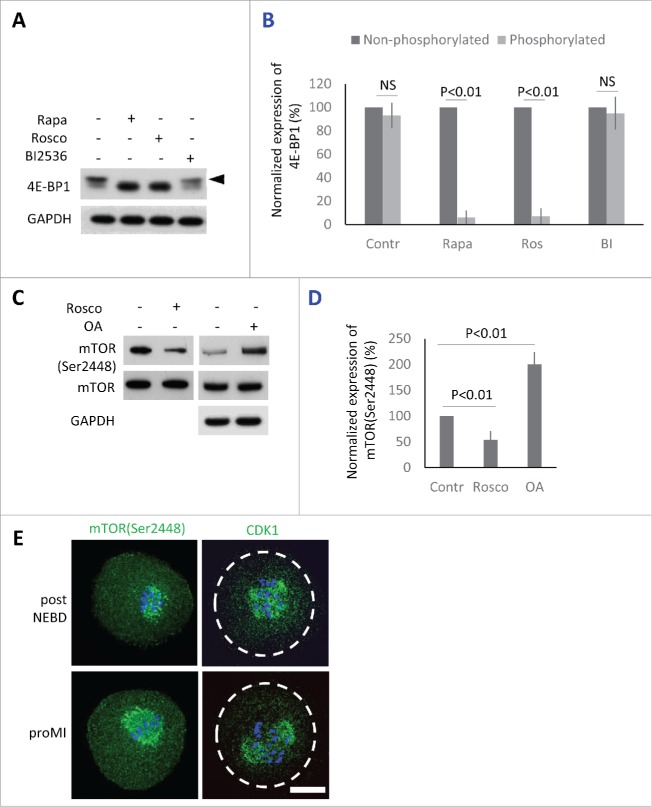
Protein kinases phosphorylating 4E-BP1 in the oocytes. (A) Detection of 4E-BP1 by immunoblotting in the oocytes treated with specific inhibitors Rapa (100 nM), Rosco (10 µM), or BI2536 (100 nM) post-NEBD. Arrowhead marks the presence of upper band (phosphorylation shift) of 4E-BP1 in the oocytes treated for 2 h post-NEBD, GAPDH was used as a loading control, a typical experiment from at least 3 replicates is shown. (B) Quantification of non-phosphorylated and phosphorylated form of 4E-BP1 in the post NEBD oocytes. Data are presented as mean ± SD, Student's t-test, NS = non-significant. (C) CDK1 effect on mTOR phosphorylation in the oocytes treated by Rosco (10 µM) or OA (1 µM). Immunoblot was probed with mTOR(Ser2448) and control (mTOR and GAPDH) antibodies. Twenty oocytes were used per sample. (D) Presence of mTOR(Ser2448) normalized to the mTOR in the Rosco or OA treated oocytes. Data are presented as mean ± SD, Student's t-test. (E) Localization of mTOR(Ser2448) and CDK1 in the post NEBD and pro-MI stage oocytes, n ≥ 30, phospho-specific antibody (green) and DNA (blue). Scale bar = 20 µm.

Previously we have shown that suppression of mTOR activity using 100 nM mTOR inhibitor Rapamycin (Rapa) significantly represses phosphorylation of 4E-BP1, however, it does not prevent the oocytes to reach MII stage.^18^ Phosphorylation of 4E-BP1 by CDK1 kinase^32-34^ has been also described in other systems, in which it becomes activated at the onset of both mitosis,^34,35^ and meiosis.^36^ In mammalian oocytes, CDK1 activity is essential for the major morphological events occurring during meiotic maturation (including NEBD, chromosome congression and condensation, formation of the meiotic spindle) and its inhibition in the beginning of maturation results in the complete block of meiosis with oocytes arresting in the GV stage.^37^ We therefore investigated the ability of the CDK1 inhibitor 10 µM Roscovitine (Rosco), as well as 100 nM mTOR inhibitor Rapa, to suppress phosphorylation of 4E-BP1 post NEBD. Rapa or Rosco were added to the culture media 1h PIW. Similarly to Rapa, the inhibition of CDK1 also showed significant suppression of phosphorylation shift (Fig. 3A). Next, based on its activity described in mitotic cells, we decided to determine whether PLK1 is also involved in the phosphorylation of 4E-BP1.^38^ We added 100 nM specific PLK1 inhibitor BI2536^39^ to the oocytes 1h PIW. However, no effect of BI2536 on 4E-BP1 phosphorylation was seen after 2h of culture (Fig. 3A and B).

Our study supports other published research^32-34^ documenting that CDK1/CYCB1 (MPF) kinase is also involved in 4E-BP1 phosphorylation and in the inactivation of the its suppressor function. Mitosis is commonly thought to be associated with reduced cap-dependent protein translation, however, our previously published results.^4,5,9^ show that the main regulators of cap-dependent translation initiation become activated at the onset of meiosis in pig oocytes. Therefore, we elucidated whether MPF had an impact on the activation of mTOR in mouse oocytes. By downregulation of CDK1 using Rosco treatment (added post-NEBD) we found a significant decrease in phosphorylation of mTOR(Ser2448) (Fig. 3C and D). On the other hand, treatment with Okadaic Acid (OA) substantially increased phosphorylation/activation of mTOR in the treated oocytes, when compared with control oocytes (Fig. 3C and D). Our WB data revealed that MPF influenced the activity of mTOR in the mammalian oocyte after the re-initiation of meiosis. We expected a positive correlation between the localization of the kinases and that of the phosphorylated forms of 4E-BP1. The ICC experiments indeed showed that fluorescence for both mTOR(Ser2448) and CDK1 kinases are present at the newly forming spindle or bipolar spindle (Fig. 3E), which was in good agreement with the localization of phosphorylated 4E-BP1 (Fig. 2).

Reduced cap-dependent protein translation is believed to be connected with mitosis. However, Heesom et al.^32^ and Huda et al.^34^ have demonstrated that cap-dependent translation is generally sustained during mitosis and 4E-BP1 becomes phosphorylated after entry to mitosis. Thus we isolated cumulus cells (CCs) from GV and MII oocyte-cumulus complexes to investigate the phosphorylated status of 4E-BP1 in other cells that are naturally present in the G0 or G1 stage^40^ of the cell cycle. WB data from CCs lysates revealed that 4E-BP1 was not phosphorylated in the CCs isolated either from GV or from MII CCs (Supplementary Fig. 4A). Oocytes in GV and MII stages were used as a control of the protein mobility shift. Accordingly, in the ICC experiments there was no 4E-BP1 phosphorylation signal observed for Thr37/46/70 and Ser65 in the CCs, although 4E-BP1 was present in this cell type (Supplementary Fig. 4B).

**Figure 4.**
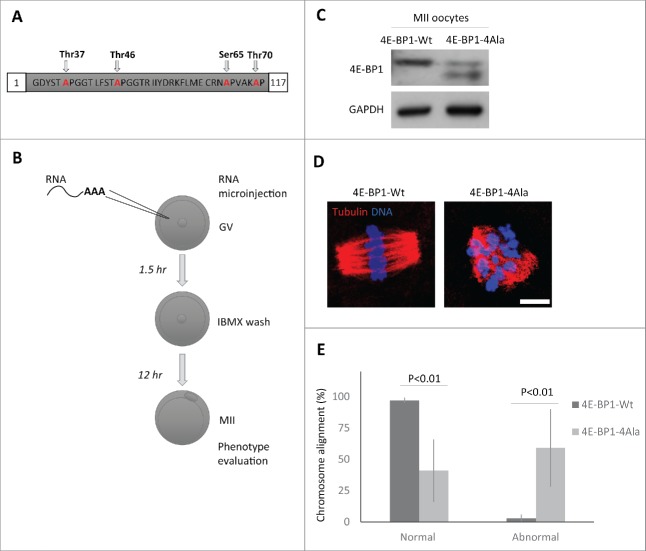
Down-regulation of 4E-BP1 phosphorylation in oocytes results in defects in the MII spindle assembly. (A) Scheme of dominant negative mutant construct of 4E-BP1–4Ala used for in vitro transcription. (B) Scheme of experimental procedure to express 4E-BP1 RNA constructs in the oocyte. (C) Immunoblotting evaluation of expression of microinjected non-phosphorylable form (marked by arrowhead) of 4E-BP1 in the matured MII oocytes n = 2. GAPDH was used as a loading control. See Figure S5. (D) Confocal images of MII spindles of oocytes microinjected with 4E-BP1-Wt or dominant negative mutant 4E-BP1–4Ala, Tubulin (red) and DNA (blue). Scale bar = 10 µm. (E) Quantification of chromosome alignment in the metaphase plate, MII oocytes expressing 4E-BP1-Wt or 4E-BP1-Ala RNA. Data are presented as mean ± SD, Student's t-test, n ≥ 25.

Altogether, our results suggest that upon exit from prophase the activity of CDK1/CYCB1 (MPF) is required for the phosphorylation of 4E-BP1, most likely via activation of mTOR.

### Expression of a dominant negative 4E-BP1 mutant promotes aberrant spindle formation

4E-BP1 phosphorylation releases eIF4E binding to permit translation initiation; the overall increase in phospho-4E-BP1 in the cytoplasm may facilitate maternal mRNA translational recruitment in the cytoplasm. To down-regulate phosphorylation of 4E-BP1, we expressed RNA coding for 4E-BP1 with all 4 phospho-sites mutated - Thr37/46/70 and Ser65 (4E-BP1–4Ala; Fig. 4A). Microinjection (Fig. 4B) of the *in vitro* transcribed (IVT) RNA coding for 4E-BP1-wild type (4E-BP1-Wt) or 4E-BP1–4Ala showed that the whole population of endogenous and exogenous 4E-BP1-Wt was phosphorylated in the MII oocytes (Fig. 4C; also see Fig. 1A and D), however, in MII oocytes microinjected with *4E-BP1–4Ala* RNA 2 not phosphorylated bands were present (depicted by arrowhead) and upper band of phosphorylated endogenous 4E-BP1 (Fig. 4C). Moreover, ICC detection with 4E-BP1 antibody in the microinjected oocytes showed significant increase of the intensity of the 4E-BP1 protein level for both injected constructs in comparison with no injected group (Supplementary Fig. 5; mean value ± 29 % in the 4E-BP1-Wt and mean value ± 23 % in the 4E-BP1–4Ala, *P*<0.001 Student's *t*-test). Microinjected oocytes with *4E-BP1–4Ala* RNA extruded a polar body, however, ICC analysis showed significant increase in aberrant spindles accompanied by the absence of chromosome alignment to the metaphase plate in 59% of the 4E-BP1–4Ala injected oocytes (mean value ± 31%; *P*<0.01, Student's *t*-test), whereas only 3% of the 4E-BP1-Wt injected oocytes produced them (mean value ± 2 %; *P* < 0.01, Student's *t*-test, Fig. 4E).

**Figure 5.**
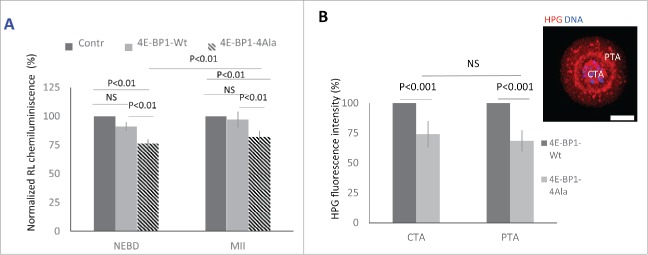
4E-BP1 effects on protein synthesis in the oocytes. (A) Renilla luciferase reporter carrying 5′UTR TOP motive of *Eef2* co-injected with *4E-BP1-Wt* or *4E-BP1-Ala* RNA. In the control no *4E-BP1* RNA was used and the IRES motive Firefly Luciferase was used as a loading control. Chemiluminescence was measured in the post NEBD stage (mean value ± 6 and 4 %, Student's t-test, NS = non-significant) and MII stage oocytes (mean values ± SD, Student's t-test). Data are presented as mean ± SD, n ≥ 10 replicates. (B) Measurement of *in situ* translation intensity in the chromosomal area (CTA, mean value ± SD, Student's t-test, NS = non-significant) and perispindular translational area (PTA, mean value ± SD, Student's t-test) in the post NEBD oocytes, HPG (red) and DNA (blue). Data are presented as mean ± SD, n ≥ 21. Scale bar = 20 µm.

It is accepted that 4E-BP1 is a key player in cap-dependent translation^27^ which predominantly utilizes mRNA with TOP motif.^41^ To further investigate this, we examined the expression of a dominant negative mutant of 4E-BP1^41^ and its influence on the translation of the *Renilla* Luciferase (RL) reporter with canonical TOP motive of the *Eef2*^41^. We performed microinjection of IVT RNA coding 4E-BP1-Wt or 4E-BP1–4Ala with RL reporter RNA and *Firefly* Luciferase (FL) with IRES motif as a microinjection loading control. Analysis of chemiluminescence showed a significant decrease of RL expression in the oocytes expressing 4E-BP1–4Ala in comparison with the control injected with RL and FL (Fig. 5A). Decrease of RL expression was non-significant in 4E-BP1-Wt RNA injected (mean value ± 6%, *P* > 0.05, Student's *t*-test) in the post-NEBD stage in comparison with 24% significant decrease in the *4E-BP1–4Ala* RNA injected groups (mean value ± 4%, *P*<0.01, Student's *t*-test). RL expression in the MII oocytes showed significant (18%) decrease in the oocytes injected with 4E-BP1–4Ala RNA in comparison with the control group (mean value ± 5%, *P* < 0.01, Student's *t*-test). Moreover, we analyzed in situ translation (Fig. 5B) in the 2 distinct areas of the oocyte after expression of 4E-BP1-Wt or 4E-BP1–4Ala, one at the area of the newly forming spindle (Chromosomal Translational Area; CTA) and the second at the Perispindular Translational Area (PTA). We detected a significant decrease of translation at the CTA (26 %, mean ± 11 %; *P* < 0.01, Student's *t*-test) and PTA (32 %, mean ± 9 %; *P* < 0.01, Student's *t*-test), however, without significant differences between CTA and PTA (*P* > 0.05; Fig. 5B).

### Discussion

Here we present an analysis of regulation of 4E-BP1 phosphorylation during meiotic division of the mammalian oocyte, a cell that naturally undergoes NEBD, then enters prometaphase and resumes meiosis further by asymmetric cytokinesis creating a fertilizable egg and a polar body. The progress of oocytes through cell cycle is highly synchronized, with rapid inactivation/phosphorylation of 4E-BP1, which suggests that cap-dependent translation is highly active in this cell type and stage.

In accordance with Mayer et al.^23^ we were not able to detect 4E-BP2 and 4E-BP3 proteins suggesting that 4E-BP1 is the only form of eIF4E-binding protein present in mouse and bovine oocytes. However, mRNAs coding all 3 isoforms are present and stable in mouse oocytes during maturation indicating their role post-fertilization during early embryonic development, or alternatively, they might be translated to substitute 4E-BP1 in case of an insufficiency of the 4E-BP1 form.^42^

Here we show that the main effector kinases of 4E-BP1 phosphorylation are mTOR and CDK1, which become highly active after the resumption of meiosis both in mouse, human and, also bovine oocytes (mTOR,^9,23^ and MPF^36^), which is similar to mitosis.^43^ It has been reported that also PLK1 promotes phosphorylation of 4E-BP1 in mitotic cells,^44^ however, inhibition of PLK1 in mammalian oocytes did not show any effect on 4E-BP1 phosphorylation in our model system. Inhibition of mTOR or CDK1, on the other hand, strongly affects 4E-BP1 phosphorylation in a very similar manner. These findings suggest the existence of a different mechanism of 4E-BP1 phosphorylation in the meiotic cell. We further show that inhibition of CDK1 kinase activity results in inhibition of mTOR phosphorylation on the site activation (Ser2448), suggesting that CDK1 exerts its effect on 4E-BP1 phosphorylation via activation of mTOR, although we cannot exclude the possibility that CDK1 phosphorylates 4E-BP1 directly. So, in accordance with Heesom et al.^32^ we show that the main regulator of 4E-BP1 phosphorylation in mouse oocytes is mTOR, on the other hand, CDK1 activity is in our system required for the full mTOR activation rather than for direct 4E-BP1 phosphorylation. It is known that mTOR is phosphorylated and activated in mitotic cells by AKT,^45^ however, according to our results, it seems that during mammalian meiosis this pathway is not sufficient for full mTOR activation, which is likely to be mediated by CDK1.

An increase in 4E-BP1 phosphorylation has been previously seen in porcine, bovine and mouse oocytes,^4,8,9,21,22^ however, only recently the localization of the differently phosphorylated forms of 4E-BP1 has been described in mouse oocytes.^38^ The nature and role of Ser65 and Thr70 phosphorylation for spindle localization is unclear at the present time, although it should be noted that cap-dependent translation becomes elevated at the onset of meiosis and is inactivated later when it exits meiosis (fertilization).^6^ Romasko et al.^22^ show that 4E-BP1(Ser112) has similar localization as 4E-BP1(Ser65) in our study. Regulation of 4E-BP1 phosphorylation at the spindle is likely to be temporally and mechanistically distinct from its regulation in the rest of the oocyte. The dynamic spatial and temporal pattern of localization of phosphorylated 4E-BP1 that forms at the spindle is indicative of a novel mechanism promoting localized protein production related to transcripts localized at the spindle. Depolymerization of the newly forming spindle by Noco treatment changed the 4E-BP1(Thr70) pattern, however, phosphorylation still persisted at the chromosomal area. This suggests the existence of a mechanism, which maintains phosphorylation at this position, most likely involving Lamin A/C and/or endoplasmic reticulum structures surrounding the spindle assembly area. Such a mechanism would promote the accumulation of specific proteins by microtubule-independent machinery, involving some sort of semipermeable membrane^46^ formed from microfilaments,^47,48^ ER,^49-51^ LMN^9^ and possibly other constituents.

A number of studies,^22,52-57^ have reported the enrichment of specific mRNAs at the spindle, which may contribute to the local proteome. Beside the enrichment of global translation at the oocyte spindle,^9^ Romansko et al.^22^ has also shown that *Mis18a* mRNA coding MIS18 Kinetochore Protein A is localized at the oocyte spindle, which is required for metaphase alignment and proper chromosome segregation.^58^ Another example of localized translation has been documented by Bomar et al.^52^ who identified the localization of *Akap95* (A kinase-anchoring protein) mRNA at the MII spindle without protein expression at this stage, but the mRNA was then translated after fertilization and the protein was present in the female pronucleus causing an unequal distribution between maternal and paternal nuclei in the zygote. Local transcriptome coupled with its translation suggests the role of translational machineries, where mTOR, CDK1 and 4E-BP1 are key players, the mechanism that is used by meiotic and mitotic cells of various species. However, differences between the cell types suggest there are distinct modes of regulation.

There are various factors involved in spindle formation. Apart from the specific transport of mRNA to the spindle, a population of RNA might already be present in the nucleus,^9,56,59,60^ which indicates a significant contribution of the local transcriptome to the formation of spindle directly post-NEBD. In accordance with this, 4E-BP1 is enriched in the nucleus in its non-phosphorylated state. 4E-BP1 in the nucleus might by bound to the 5′UTR of mRNAs, where it probably functions as a translational repressor. Consequently, after its hyperphosphorylation following NEBD, it becomes inactivated and in such a way promotes the translation of specific mRNAs at the newly forming spindle. These results suggest that the function of mRNA retention in the nucleus may be to sustain translational repression, and that their subsequent translation can be regulated in a spatiotemporally restricted manner in response to cell cycle events.

We propose that meiotic phosphorylation of 4E-BP1 on Ser65 and Thr70 by mTOR acts to stimulate cap-dependent translation as the oocyte proceeds though meiosis (particularly after NEBD) and that specific localization of the key cap-dependent translation regulatory factors,^22,61^ is essential for the translation of specific mRNAs at the spindle area to ensure errorless meiotic progression. We identify the 2 kinases mTOR and CDK1 involved in the inactivation of the 4E-BP1 at the spindle where all the important regulators are present. Using PLK1 inhibitor BI2536 we show that PLK1 kinase is not involved in 4E-BP1 phosphorylation in mouse oocytes and also, that CDK1 exerts its influence via the phosphorylation (and as such further activation) of mTOR, which as a result is likely to phosphorylate Ser65 and Thr70 of 4E-BP1. However, we cannot exclude the possibility that CDK1 phosphorylates at least one of these sites directly, as was previously reported by Heesom et al.^32^ and Shuda et al.^34^ Since the effect of CDK1 inhibition on the level of 4E-BP1 phosphorylation is less pronounced in later stages of meiosis (data not shown) it is tempting to speculate that the increased activation of mTOR mediated by CDK1 might be temporally and possibly also spatially restricted to the most critical process during early meiosis, i.e. formation of the meiotic spindle. Such hypothesis is supported also by the data obtained by us and other studies^38^ showing the increased presence of 4E-BP1 phosphorylated forms at the spindle and in the chromosomal area. It is also interesting to note that CDK1 has been shown to directly phosphorylate the key mTOR binding partner Raptor during mitosis.^62^ This reinforces our conclusions and those from other studies suggesting that mTOR activity is highly regulated by cell cycle progression. A number of other proteins involved in the regulation of translation have also been described previously. Papst^63^ reported that Ribosomal Protein S6 Kinase is a substrate for CDK1/CYCB1 in mitosis and Elongation factor-1^64^ in the *Xenopus* oocytes during meiotic cell division is a physiologic substrate of CDK1/CYCB1 in mitosis.

After fertilization when the nuclear envelope is reformed again at the end of meiosis, phosphorylation of 4E-BP1 disappears.^5,9^ This indicates a specific/exclusive role of this pathway in meiotic maturation, which is also supported by our findings showing that no phosphorylated 4E-BP1 is present in the CCs, naturally occurring in the G0 or G1 stage. We might conclude that phosphorylation of 4E-BP1 follows exit from prophase of the cell cycle. It has been reported previously that overall protein synthesis becomes reduced during meiosis.^4,9,65^ However, studies in synchronized HeLa cells have shown that this inhibition ceases by late telophase^66^ and that overall protein synthesis increases rapidly as cells enter G1-phase.^67^

Here we show that the presence of a non-phosphorylated 4E-BP1 population in an oocyte that progresses through meiosis results in aberrant morphology of the metaphase II spindle that is most likely the result of impaired translation of a subset of RNAs. Previously, we have described the effect of mTOR/4F pathway downregulation on in situ translation at the chromosomal area.^9^ Our current finding shows that a non-phosphorylated mutant does not display significant differences in the level of translation between the chromosomal and perispindular areas. This might be explained by the fact that exogenous 4E-BP1, which is loaded to the cytoplasm in the form of RNA, and its consequent 4E-BP1 protein, lacks endogenous localization in this large cell and so influences both translational areas within the cell. On the other hand, the expression of a mutant in the cytoplasm which is unable to be phosphorylated leads to downregulation of translation in the cytoplasm and at the chromosomal area.

4E-BP1 null mice are viable and fertile.^42^ However, we have observed aberrant spindle formation in the MII oocytes expressing a non*-*phosphorylatable 4E-BP1 form, which might suggest that the role of 4E-BP1 is rather in the fine tuning of meiotic progression. Regulation of 4E-BP1 in the oocyte might be affected by cell stress or by the age of the female. Moreover, insulin stimulates the mTOR signaling pathway^68^ and insulin signaling promotes the production of high-quality oocytes.^69^ Consistently, oocytes from diabetic mice display spindle abnormalities, which can be reversed by pancreatic islet transplantation.^70^ Our findings showing localization of phosphorylated/inactivated 4E-BP1 at the spindle also suggest the existence of a mechanism that links maternal age and environmental exposures to diminished oocyte quality arising from defective spindle formation and function. We show that mTOR becomes also activated post NEBD in the human oocyte, with strong signal at midbody in the MII oocyte, suggesting its similar role in the human oocyte meiosis in specific translational regulation, as it plays in the mouse oocyte. Here, mTOR pathway might contribute to the age related chromosome segregation errors in the woman oocytes, similarly as it has been documented in the mouse model,^9^ as well as in mammalian and yeast cells.^71^ Lapasset et al.^7^ showed that the treatment with Rapa resulted in the prevention of extrusion of second polar body in starfish oocytes. They present the absence of eIF4E dissociation from 4E-BP in the presence of Rapa without the effect on translation of Cyclin B1 or Mos. Taken together, mTOR involvement is indispensable for inactivation of translational repressor 4E-BP1, which prevents the synthesis of essential proteins necessary for a correct completion of the meiotic and mitotic divisions. In addition to translational initiation factors, Ribosomal protein S3 (RPS3) is present at the mitotic^72^ or newly forming meiotic spindle.^73^ RPS3 knockdown causes arrest in mitotic metaphase,^72^ which resembles the effect of mTOR inhibition^23^ in the bovine oocyte. The influence of known effector kinases in the inactivation of the translational repressor 4E-BP1 might be essential for the temporal and spatial translation of specific mRNAs at the spindle area to ensure errorless meiotic progression.

In this study we propose that localized translational regulation at the oocyte spindle regulated though an mTOR/CDK1 pathway might represent a mechanism which links spindle formation and function with the temporal and spatial regulation of the local transcriptome in the particular subcellular areas, which affects oocyte quality. There is still much to learn about the dynamics of distribution of mRNA and translational regulatory components, as well as how exactly these are regulated in the different cellular compartments. Further elucidation of the relationship between cytoskeletal elements and translation machinery may help to explain the logistics of translational control of spindle assembly and chromosome segregation.

## Material and methods

### Oocytes isolation and maturation

Mouse ovaries were obtained from CD1 mice at least 6 weeks old which were stimulated to by intraperitoneal injection of 5 UI of pregnant mare serum gonadotropin (PMSG; Folligon, Merck Animal Health) 46 h before collection. GV oocytes were isolated into transfer medium Tetkova et al.^74^ supplemented with 100 µM of 3-isobutyl-1-methylxanthine (IBMX) used to prevent spontaneous resumption of meiosis. Selected oocytes were stripped of the cumulus cells and cultured in M16 medium (Millipore) without IBMX at 37°C, 5% CO_2_. After 70 min post IBMX wash (PIW) at least 90% of oocytes underwent nuclear envelope breakdown (NEBD, resumption of meiosis; G2/M transition) and oocytes arrested in the GV were discarded. Pro-metaphase I (pro-MI) and metaphase I (MI) stage oocytes were collected after post IBMX wash at 3 h (post-NEBD), 7 h (pro-MI) and 12 h (MII). All animal work was conducted according to Act No 246/1992 on the protection of animals against cruelty. Human oocytes, not used in human reproduction, were obtained from the Obstetrics and Gynecology Clinic of the General University Hospital in Prague. The project was accredited (#30/12) by the Ethical Committee of the General Hospital, Prague.

### Oocyte treatments

Mouse oocytes were treated with 100nM BI2536 for 2 hours post NEBD (Axon Medchen), 1 µM Nocodazole for 1 h (M1404, Sigma-Aldrich), 100 nM Rapamycin (#9904, CST) or 10 µM Roscovitine (R7772, Sigma-Aldrich); 1 µM Okadaic acid (OA, CAS 459616, Millipore) for 2 h after NEBD. For nascent protein synthesis specific stage NEBD-2 h, oocytes were cultured in methionine-free medium (Gibco) supplemented with 1% dialyzed fetal bovine serum (10,000MW; Sigma) and 50 mM L-homopropargylglycine (HPG) for 30 min. HPG was detected by using a Click-iT Cell Reaction Kit (Life Technologies). *In situ* translation detection showed increased incorporation of HPG in the chromosomal area (CTA) and perispindular area (PTA^9^)

### RNA isolation and quantitative RT-PCR

RNA was extracted with RNeasy Plus Micro kit (Qiagen) according to manufacturer's instructions. Genomic DNA was depleted using guide columns. H_2_O for qRT-PCR was used for RNA elution in amount of 25 µL for 25 oocytes. Samples were stored at −80 °C until expression analysis. mRNA equivalent for 1 oocyte was amplified by a One-step RT-PCR kit (Qiagen) with real-time detection using SybrGreenI fluorescent dye on a Rotor Gene 3000 instrument (Corbett Research, Australia). The qRT-PCR reactions were prepared in duplicates in one run. Reaction conditions were: reverse transcription at 50°C for 30 min, initial activation at 95°C for 15 min, cycling: denaturation at 95°C for 20 sec, annealing at a temperature specific for each set of primers (see Table S1) for 20 sec, extension at 7°C for 30 sec. Products were verified by melting analysis and gel electrophoresis on 1.2% agarose gel with ethidium bromide staining. The relative concentration of templates in different samples was determined using comparative analysis software (Corbett Research). The results for individual target genes were normalized according to the relative internal standard GAPDH. The data are presented from at least 3 biologic replicates. The significant differences between GV and MII were evaluated using *t*-test (PrismaGraph5).

### Immunocytochemistry

Mouse and human oocytes were fixed for 20 min in 4% PFA in phosphate saline buffer (PBS). Oocytes were permeabilized for 10 min in 0.2% Triton X-100 in PBS, then washed with PVA/PBS. Oocytes were incubated with primary antibodies at 4°C overnight. We are using human 4E-BP1 nomenclature to unify the text discussing human and mouse systems. The human 4E-BP1 sequence of amino acid numbers is greater by one. The following antibodies were used in 1:100 dilution: rabbit anti-4E-BP1 (#9452, CST), rabbit anti-phospho-4E-BP1(Thr70) (#13396, CST), rabbit anti-phospho-4E-BP1(T37/46) (#9459, CST), rabbit anti-phospho-4E-BP1(Ser65) (#9451, CST), rabbit anti-CDK1 (#9112, CST), mouse anti-tubulin (#T6793, Sigma) and γ-tubulin (#T6557, Sigma), rabbit anti-phospho-mTOR(Ser2448, #2971, CST) and mouse anti-LMNA/C (SAB4200236, Sigma Aldrich). After washing in PBS, detection of the primary antibodies was performed by cultivation of the oocytes with relevant Alexa Fluor 488, 594 or 647 conjugates (diluted 1: 250) for 1 h at room temperature. Oocytes were then washed 2 times for 15 min in PVA/PBS and mounted using Vectashield Mounting Medium with DAPI (H-1200, Vector Laboratories). Samples were visualized using a Leica SP5 inverted confocal microscope (Leica Microsystems) in 16 bit depth. Images were assembled in LEICA LasAFX (Leica Microsystems) software and equatorial sections were quantified by Image J software (http://rsbweb.nih.gov/ij/).

### Western blot

Oocytes were lysed with 6 µl of Millipore H_2_O and 2, 5 µl of 4x lithium dodecyl sulfate, sample buffer NP 0007 and 1 µl reduction buffer NP 0004 (Novex, Thermo Fisher Scientific) and boiled at 100°C for 5 min. If not stated otherwise, sample of 50 oocytes per sample was used. To detect phosphorylation shift, oocytes were dissolved in the 20 µl of the 1x NEBuffer with 800 U of LPP enzyme (P0753, New England BioLabs) and incubated overnight at 30°C, LPP was omitted in the control sample (LPP-). Lysates were separated using a 4–12% gradient polyacrylamide gel SDS (NP323BOX, Life Technologies) page and transferred to an immobilon P membrane (PVDF; Millipore) using semidry blotting system (Biometra GmbH). Membranes were blocked for 1 h, in 1–5% skimmed milk dissolved in Tween-Tris-buffer saline (TTBS, pH 7,4) according to antibody (list of primary antibodies and dilutions is below). After 3 cycles for 10 min washing in TTBS, membranes were incubated at 4°C overnight in 1% milk/TTBS with the following primary antibodies: GAPDH (rabbit, G9545, Sigma-Aldrich) and Tubulin (mouse, T6793, Sigma-Aldrich) antibodies were diluted 1:30 000 and 4E-BP1 (rabbit, 9452, CST), 4E-BP1(T69) (rabbit, 9455S, CST), 4E-BP1(T36/45) (rabbit, 9459, CST), 4E-BP1(S64) (rabbit, 9451S, CST), anti HA (rabbit, 3724, CST) antibodies were diluted 1:500; mTOR(Ser2448) (rabbit, 2971S, CST), mTOR (rabbit, 2972, CST) antibodies were diluted 1:8 000 and 1:2 000 respectively. After 3 cycles of 10 min washing in TTBS the membrane was incubated for 1 h with secondary antibody Peroxidase Anti-Rabbit Donkey (711–035–152, Jackson immunoresearch) or Peroxidase Anti-mouse Donkey (715–035–151, Jackson immunoresearch) in 1:7.500 dilution in 1% milk/TTBS 1 h at room temperature. Immunodetected proteins were visualized by ECL (Amersham, GE Healthcare life science), films were scanned using a GS-800 calibrated densitometer (Bio-Rad) and quantified using Image J software (http://rsbweb.nih.gov/ij/).

### Microinjection

GV stage mouse oocytes were microinjected in transfer medium with IMBX on an inverted microscope Leica DMI 6000B with Transferman NK2 and Femtojet (Eppendorf). Oocytes were injected with *in vitro* transcribed RNA (mMessage, Ambion) from mutant plasmid pCW57.1–4E-BP1–4Ala^41^ and pCMV3-N-HA-4E-BP1 (generous gift of professor Nahum Sonenberg, McGill University, Montreal, Canada; Gingras et al.^27^). Approximately 5 pl of RNA solutions of 4E-BP1-Ala or 4E-BP1-Wt diluted in RNAse free water, to concentration 50 ng/µl were microinjected into oocytes.

### Dual-luciferase assay

Oocytes were injected in the presence of IBMX with 50 ng/µl of IVT RNA (mMessage, Ambion) from *Renilla Luciferase* constructs (*Eef2*–5′UTR - RL; #38235; Addgene) with combination of injection amount control *Firefly Luciferase* (FL; #18964; Addgene^75^) and RNA for 4E-BP1-Wt or 4E-BP1–4Ala in the presence of IBMX. Oocytes were cultured for 5 h without IBMX. At least 5 oocytes were lysed in 5 µl of Passive Lysis Buffer and stored at −80°C until measurement of chemiluminescence by Dual-Luciferase Assay System (Promega) according to the manufacturer's instructions. Signal intensities were measured using a Glomax Luminometer (Promega). Activity of RL was normalized to the FL luciferase.

### Statistical analysis

Experiments were repeated at least 3 times unless stated. Mean and SD values were calculated using MS Excel, statistical significance of the differences between the groups was tested using Student's *t*-test (PrismaGraph5) and *P*<0.05 was considered as statistically significant.

## Supplementary Material

1295178_Supplemental_Material.pdf

## Abbreviations

Akap95: Kinase (PRKA) anchor protein 8
CCs: Cumulus cells
CDK1: Cyclin dependent kinase 1
CTA: Chromosomal translational area
CYCB1: Cyclin B1
DAPI: 4', 6-diamidino-2-phenylindole
ER: Endoplasmic reticulum
eIF4E: Eukaryotic initiation factor 4E
eIF4G1: Eukaryotic initiation factor 4G1
eIF4A: Eukaryotic initiation factor 4A
FL: Firefly Luciferase
FRAP kinase: FKBP-12-rapamycin-associated protein
G1-phase: Gap 1 phase
GV: Germinal vesical stage
HPG: L-homopropargylglycine
HA: Hemagglutinin
IBMX: 3-Isobutyl-1-methylxanthine
IRES: Internal ribosome entry site
IVT: *In vitro* transcribed
LMN: Lamin A/C
Mis18a: MIS18 kinetochore protein A
mTOR: Mammalian target of rapamycin
MI: Metaphase of first meiotic maturation
MII: Metaphase of second meiotic maturation
MPF: Maturation promoting factor
Noco: Nocodazole
NEBD: Nuclear envelope breakdown
OA: Okadaic acid
PTA: Perispindular translational area
PVA: Polyvinyl alcohol
PIW: Post IBMX wash
PLK1: Polo-like kinase1
PKB/ AKT: Protein kinase B/ serine/threonine-specific protein kinase
PBE: Polar body extrusion.
Ras/Raf/ERK pathway: Mitogen-activated protein kinases pathway
Rosco: Roscovitine
RL: Renilla Luciferase
Rapa: Rapamycin
S6 kinase: Ribosomal s6 kinase
TOP: Terminal oligopyrimidine motif
3´UTR: Three prime untranslated region
4E-BP1: Eukaryotic translation initiation factor 4E-binding protein 1
4E-BP2: Eukaryotic translation initiation factor 4E-binding protein 2
4E-BP3: Eukaryotic translation initiation factor 4E-binding protein 3
5´UTR: Five prime untranslated region
