# Supplementary material for: Regulation of 4E-BP1 activity in the mammalian oocyte
Source: Cell Cycle. 2017 Mar 8;16(10):927–39. doi: 10.1080/15384101.2017.1295178 (PMC5462087; doi:10.1080/15384101.2017.1295178)
Supplement: 1295178_Supplemental_Material.pdf [file kccy-16-10-1295178-s001.pdf]

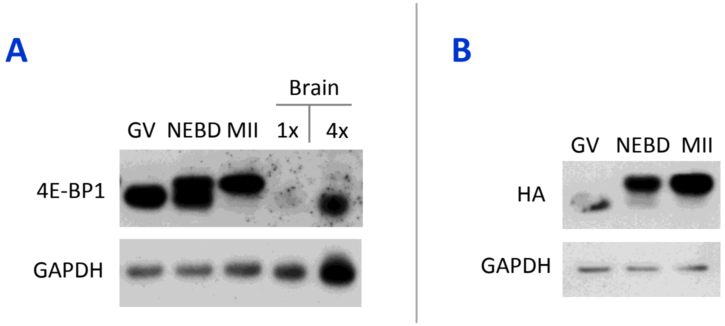

Supplementary Figure 1. 4E-BP1 becomes phosphorylated in the NEBD stage A) Fourfold increase of loading of lysate from brain shows strong 4E-BP1 product on the WB. B) Expression of microinjected RNA coding HA tagged 4E-BP1 shows shift in the NEBD and MII stages. Oocytes samples were probed with HA antibody, a typical experiment from 2 replicates is shown.

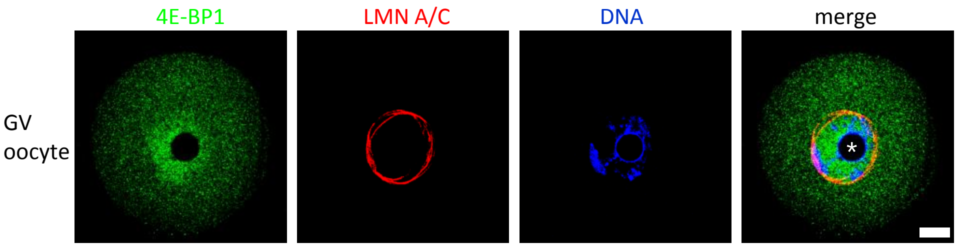

Supplementary Figure 2. GV oocyte shows strong signal of 4EBP1 in the nucleus. Confocal image of GV oocyte probed with pan-4E-BP1 (green) and Lamin A/C (LMN A/C, red) as a nuclear membrane marker and DNA (blue). Asterisk marks the nucleolus. Scale bar = 10 µm.

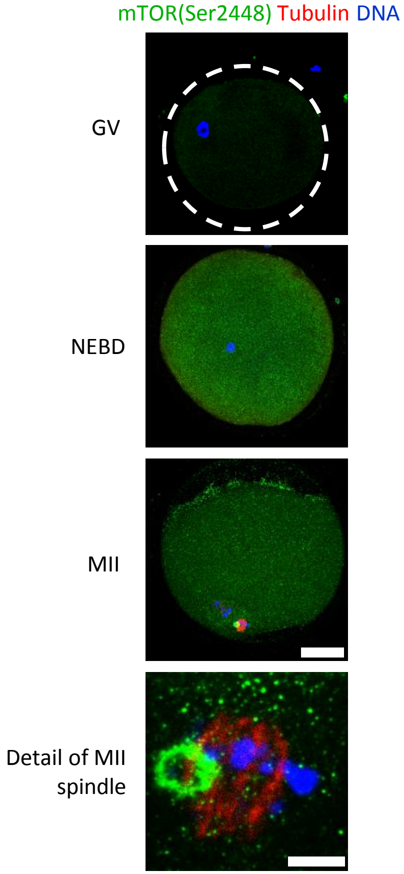

Supplementary Figure 3. Localization of mTOR (Ser2448) in human oocytes. Confocal images of human GV, NEBD and MII oocytes probed with mTOR(Ser2448; green) and Tubulin (red) antibodies by immunocytochemistry, DNA (blue). Magnified image is shown in the lowest panel to highlight the presence of mTOR(Ser2448) at the midbody structure within the MII oocyte. White line indicates oocyte edge, n≥2. Scale bars = 30 µm (whole oocyte) and 7 µm (detail).

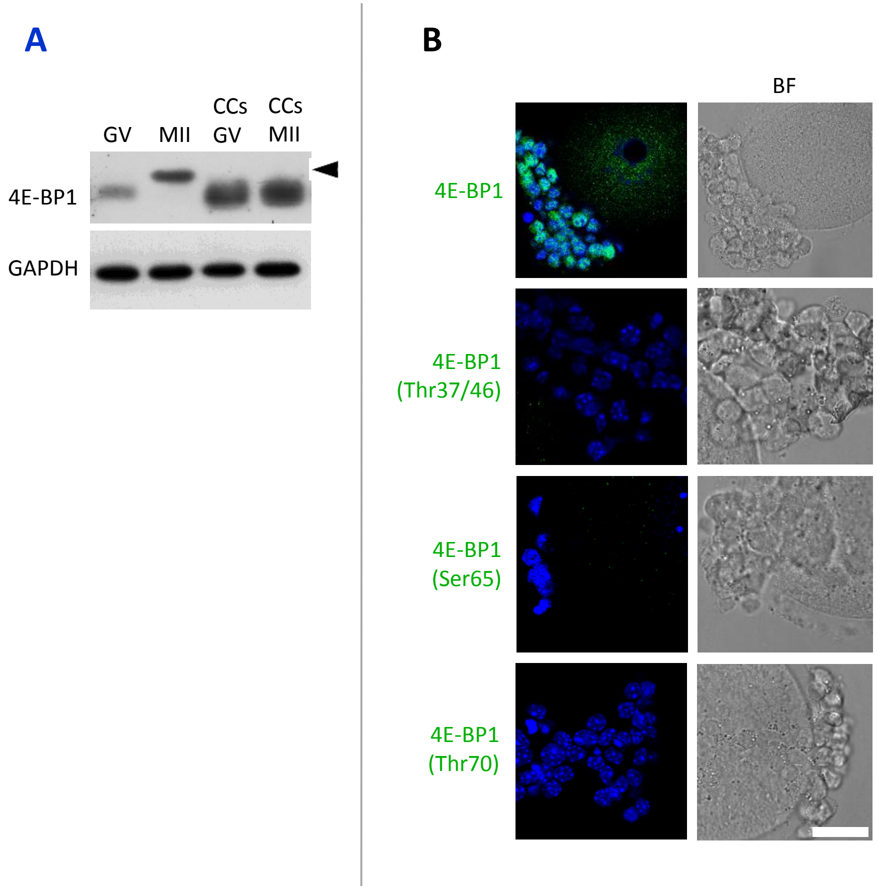

Supplementary Figure 4. Expression of 4E-BP1 and its phosphorylated forms in interphase somatic cells. (A) Immunoblot of control GV and MII oocytes and cumulus cells (CCs) isolated from GV and MII oocyte-cumulus complexes probed with 4E-BP1 and GAPDH (as a loading control), a typical experiment from 3 replicates is shown. Arrowhead marks phosphorylation shift of the 4E-BP1. (B) Oocyte cumulus cell complexes were stained with pan-4E-BP1 or phospho-specific 4E-BP1 antibodies (green) and visualized by confocal microscopy, n≥20. DNA (blue), BF (bright field). Scale bar = 20 µm.

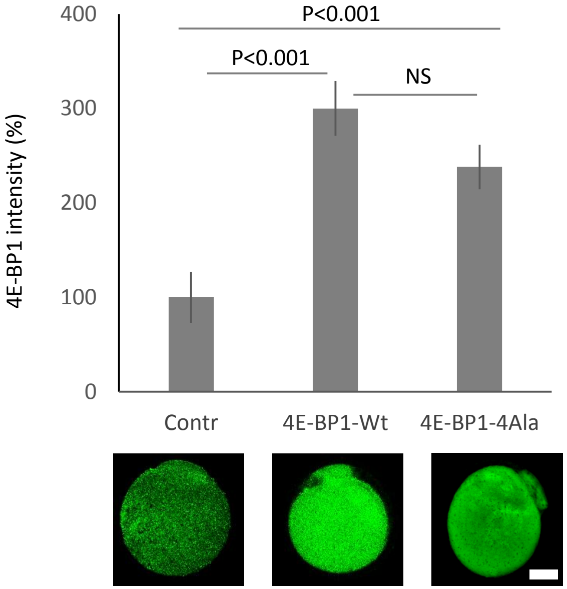

Supplementary Figure 5. Microinjection of the RNA coding 4E-BP1-Wt or 4E-BP1-4Ala shows significant increase of immunofluorescence signal of overexpressed exogenous 4E-BP1. Below, examples of immunofluorescence of 4E-BP1 in the control and injected oocytes. Data are presented as mean ± SD, Student's t-test, n≥14. Scale bar = 20 μm.

Offical symbol (Gene)	Foward 5´-3´	Reverse 5´-3´	Gene Bank ID	product size (bp)	Annealing temperature (°C)
Eif4ebp1	CATCATTGCGTCCTACGGCT	GTGTCGGAACTCACCTGTGG	NM_007918.3	201	57
Eif4ebp2	TCCCCTCTGGGTGCCAAATA	GCTTGGAGACTGCCCTAGAC	NM_010124.2	224	57
Eif4ebp3	TTCCTGCTGCTATGTCCTCGT	CCAGCAGGAACTTTCTGTCGT	NM_201256.4	150	58
Gapdh	TGGAGAAACCTGCCAAGTATG	GGTCCTCAGTGTAGCCCAAG	XM_001476707.3	93	58

Supplementary Table 1 Primers used for quantitativeRT-PCR.
